# Efficient Storing Energy Harvested by Triboelectric Nanogenerators Using a Safe and Durable All‐Solid‐State Sodium‐Ion Battery

**DOI:** 10.1002/advs.201700072

**Published:** 2017-04-18

**Authors:** Huidan Hou, Qingkai Xu, Yaokun Pang, Lei Li, Jiulin Wang, Chi Zhang, Chunwen Sun

**Affiliations:** ^1^ Beijing Institute of Nanoenergy and Nanosystems, Chinese Academy of Sciences National Center for Nanoscience and Technology (NCNST) Beijing 100083 China; ^2^ School of Chemistry and Chemical Engineering Shanghai Jiaotong University Shanghai 200240 China

**Keywords:** all‐solid‐state Na‐ion battery, energy efficiency, long‐life, pulsed output, triboelectric nanogenerator

## Abstract

Storing energy harvested by triboelectric nanogenerators (TENGs) from ambient mechanical motion is still a great challenge for achieving low‐cost and environmental benign power sources. Here, an all‐solid‐state Na‐ion battery with safe and durable performance used for efficient storing pulsed energy harvested by the TENG is demonstrated. The solid‐state sodium‐ion batteries are charged by galvanostatic mode and pulse mode with the TENG, respectively. The all‐solid‐state sodium‐ion battery displays excellent cyclic performance up to 1000 cycles with a capacity retention of about 85% even at a high charge and discharge current density of 48 mA g^−1^. When charged by the TENG, an energy conversion efficiency of 62.3% is demonstrated. The integration of TENGs with the safe and durable all‐solid‐state sodium‐ion batteries is potential for providing more stable power output for self‐powered systems.

With the large consumption of fossil energy and the resultant environmental concerns, it is imperative to develop clean energy technology. In recent years, various mechanisms and prototype devices of mechanical energy harvesting have been reported, including electrostatic and triboelectric effects,^1^, ^2^, ^3^, ^4^, ^5^, ^6^, ^7^ electromagnetic effect,^8^ and piezoelectric effect.^9^, ^10^, ^11^, ^12^, ^13^ In particular, the triboelectric nanogenerators (TENGs) with a working coupling mechanism between the triboelectric effect and electrostatic induction have been successfully demonstrated by Wang and co‐workers.^14^, ^15^, ^16^ The TENGs can be used to harvest various kinds of mechanical energy such as wind, raindrops energy,^17^ and air‐flow energy.^18^ However, the energy harvested by TENGs cannot be directly used to provide stable power output subsequently because of the randomness of mechanical energy source and pulsed alternating current (AC) output.^19^ Therefore, efficient and durable energy storage devices are highly desired to store the electrical energy harvested and converted from mechanical energy by TENGs. Although the energy generated from TENGs has been successfully stored with lithium‐ion batteries (LIBs),^20^, ^21^ there are no reports on storing the energy harvested by TENGs with a durable sodium‐ion batteries (SIBs). SIBs have attracted much interest as an alternative to lithium‐ion batteries for energy storage in the merits of the low cost and natural abundance of sodium resources.^22^, ^23^, ^24^ However, great challenges in safety, lifetime, and power density have limited scalability of conventional SIBs.^25^, ^26^, ^27^ All‐solid‐state sodium batteries contain nonflammable solid electrolytes that offer high safety compared to conventional SIBs with liquid electrolytes. All‐solid‐state sodium batteries can increase cycle life, energy density, and reduce the requirements on packaging.^28^, ^29^, ^30^, ^31^, ^32^, ^33^ Moreover, all‐solid‐state energy storage and conversion devices are especially useful for wearable applications.^34^, ^35^, ^36^ Therefore, there is a growing interest in development of all‐solid‐state sodium batteries in recent years.

Herein, we demonstrate a safe and durable all‐solid‐state Na‐ion battery with the hexagonal P2‐structure Na_0.67_Ni_0.23_Mg_0.1_Mn_0.67_O_2_ as the cathode, metallic sodium as the anode, and solid polymer electrolyte (SPE) film of perfluorinated sulfonic in the Na form (PFSA‐Na) swollen with ethylene carbonate‐propylene carbonate (EC‐PC) nonaqueous mixed solvents as the electrolyte (hereafter abbreviated as Na‐SPE). The polymer electrolyte has many merits, such as good mechanical and thermal stability, fast cation transport, as well as absence of added sodium salts.^25^ Moreover, the all‐solid‐state Na‐ion battery was demonstrated to efficiently storing the pulse electrical energy harvested by radial‐arrayed rotary TENGs. The present all‐solid‐state sodium‐ion battery has high energy densities and excellent safe properties compared with the energy storage devices usually used to couple with TENGs, like lithium‐ion batteries with liquid electrolytes and supercapacitors.

**Figure** **1**a,b shows photographs of the PFSA‐Na membrane swollen with EC‐PC mixed solvents (Na‐SPE) in folded and recovered states, respectively, indicating its flexibility nature. Figure 1c shows typical stress–strain curve of the Na‐SPE. Tensile strength, strain at break, and Young's modulus of the polymer electrolyte are 14.32 MPa, 124.8%, and 34.78 MPa, respectively. These results indicate that the present Na‐SPE polymer electrolyte membrane shows exceptional mechanical stability, which enables its direct use in solid‐state sodium batteries.

**Figure 1:**
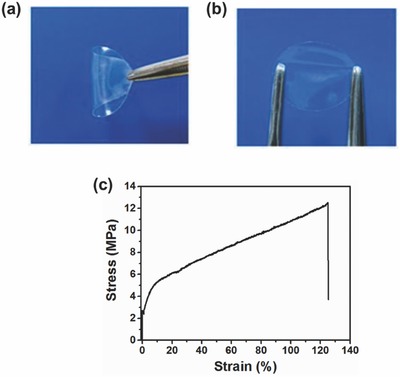
Photographs of the flexible perfluotinated sulfonic membrane in its Na form (PFSA‐Na) swollen with EC‐PC mixed solvents in a) folded and b) recovered states. c) Typical stress–strain curve of the PFSA‐Na polymer electrolyte.

Ionic conductivity of Na‐SPE polymer electrolyte was determined through two‐electrode AC impedance method. **Figure** **2**a shows the Arrhenius plot of the PFSA‐Na swollen with EC‐PC polymer electrolyte. Sodium‐ion conductivity was calculated based on the thickness of the tested electrolyte membrane and diameter of stainless electrode. The ionic conductivity of the polymer electrolyte was 2.8 × 10^−4^ S cm^−1^ at room temperature. The sodium‐ion transference number (*t*_Na+_) of Na‐SPE polymer electrolyte was determined by the Bruce–Vincern–Evans method with a symmetric Na|Na‐SPE|Na cell.^37^ After 2 h of rest time, the cell was applied with a DC voltage (5 mV) until a steady current was obtained. Then, the initial (*I*_0_) and steady (*I*_s_) currents were measured, respectively, as shown in Figure 2b. To obtain the initial (*R*_0_) and steady (*R*_s_) resistances of the electrolyte, the impedance spectra of the cell before and after the DC polarization were measured. The calculated sodium‐ion transference number of the Na‐SPE polymer electrolyte used in this work was about 0.85.

**Figure 2:**
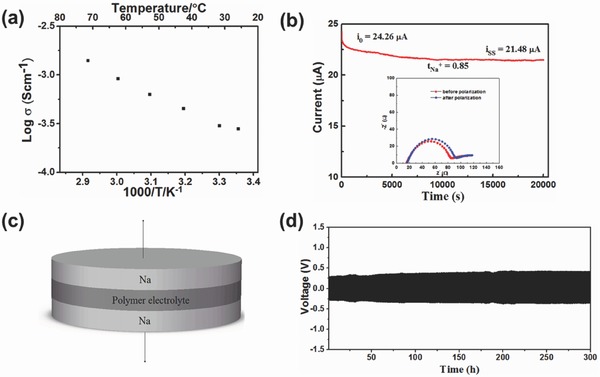
a) Arrhenius plot of the perfluotinated sulfonic membrane in its Na form swollen with the EC‐PC mixed solvent. b) Current–time profile of a symmetrical Na|Na‐SPE|Na cell after applying a DC voltage of 5 mV to the cell, used for determining Na^+^ transfer number. The inset shows the Nyquist impedance plots of the cell before and after polarization. c) Schematic of the symmetric cell for the sodium plating/stripping experiment. d) Voltage profile of the sodium plating/striping cycling with a current density of 0.2 mA cm^−2^.

The mechanical stability of the polymer electrolyte membrane against sodium dendrites was further measured in a symmetric Na|polymer electrolyte|Na cell, as shown in Figure 2c. When charging and discharging at a constant current, the sodium ions are plating/stripping from the sodium metal electrode. Figure 2d shows the voltage versus time profile of the symmetric cell over 300 h at a constant current density of 0.2 mA cm^−2^, the voltage increased from 0.3 to 0.4 V at the first 20 h and then kept stably at 0.4 V, indicating that the polymer electrolyte has an excellent mechanical stability.

Two‐electrode coin‐type cells (R2032) have also been prepared with the hexagonal P2 structure Na_0.67_Ni_0.23_Mg_0.1_Mn_0.67_O_2_ as the cathode, metallic sodium as the anode, and SPE film of PFSA‐Na as the electrolyte. **Figure** **3**a shows the charge and discharge curves of the all‐solid‐state sodium‐ion batteries at different current densities from 5 to 384 mA g^−1^ in the voltage window from 2.0 to 4.5 V. The cell shows a discharge capacity of 76 mAh g^−1^ at a current density of 5 mA g^−1^ and the voltage plateau at 3.7 V becomes shorter with increasing the current densities. Figure 3b shows the rate performance of the cell. When the charge/discharge current densities are reduced from 384 to 5 mA g^−1^, the discharge capacity can still keep ≈71 mAh g^−1^, which is about 93% of the initial discharge capacity, indicating excellent reversibility. Figure 3c,d shows the cyclic performance and coulombic efficiency of the cells at current densities of 5 and 48 mA g^−1^. The discharge capacity of the cell at a current density of 5 mA g^−1^ can still remain 70 mAh g^−1^ after 200 cycles, which is 94% of the initial discharge capacity at a current density of 5 mA g^−1^. Notably, even at a high current density of 48 mA g^−1^, the discharge capacity of the cell can still keep at 47 mAh g^−1^ after 1000 cycles, which is about 85% of the initial discharge capacity, indicating an excellent cyclability of the all‐solid‐state sodium‐ion batteries.

**Figure 3:**
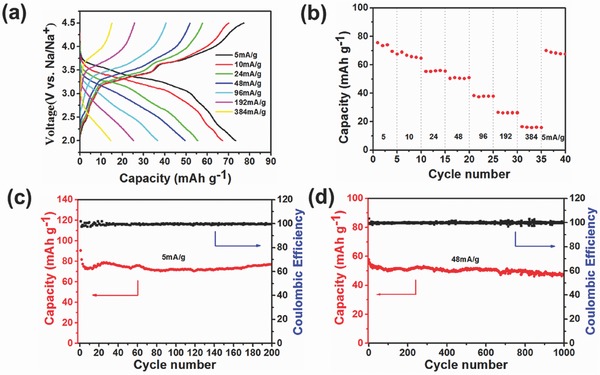
a) Charge and discharge curves of the all‐solid‐state battery at various current densities from 5 to 384 mA g^−1^; b) rate performance of Na_0.67_Ni_0.23_Mg_0.1_Mn_0.67_O_2_; and c,d) long‐term cycling performance: the capacity and Coulombic efficiency versus cycle number.

Radial‐arrayed rotary TENGs have been used to harvest mechanical energy.^38^, ^39^ The feasibility of storing pulsed electrical energy harvested by radial‐arrayed rotary TENGs was examined by this all‐solid‐state sodium‐ion battery. **Figure** **4**a,b shows schematic images of combination of the TENG, power management circuit, and an all‐solid‐state sodium‐ion battery. The energy generated from the TENG can be calculated by multiplying *I*_eq_ with the integration of the charging voltage profile (Figure 4c). As the TENG has an AC output current, the equivalent DC current value of the rectified current *I*_eq_ can be calculated approximately by the following Equation (1), 19

(1) $$I_{\mathrm{eq}}\;=\;2I_{\mathrm{peak}}/\pi$$

where the *I*_peak_ is the peak value of the rectified output current (Figure 4d). Thus, the output energy from the TENG can be calculated by the following Equation (2)

(2) $$E_{\mathrm{output}}\;=\;I_{\mathrm{eq}}\int_{0}^{t}V(t)dt$$

**Figure 4:**
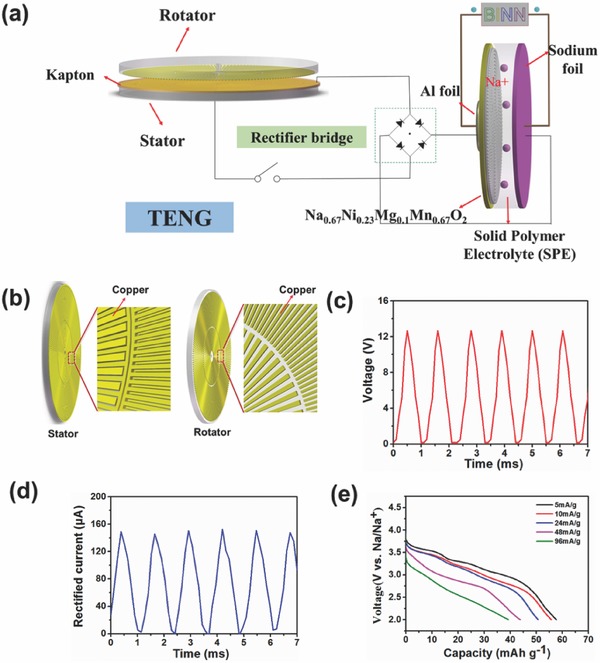
a) Schematic image of storing the pulse energy generated by the TENG in an all‐solid‐state sodium‐ion battery; b) schematic image of the partially enlarged TENG; c) output voltage and d) current of the TENG; and e) the discharge profiles of the all‐solid‐state sodium‐ion battery charged by the TENG.

The battery charged by the TENG discharges at various current densities from 5 to 96 mA g^−1^. The discharge curves of the all‐solid‐state battery are shown in Figure 4d. The all‐solid‐state sodium‐ion battery shows an initial discharge capacity of 59 mAh g^−1^ at a current density of 5 mA g^−1^ and a discharge capacity of 40 mAh g^−1^ at a current density of 96 mAg^−1^, which is comparable to the value of the batteries charged by a DC mode as shown in Figure 3a, indicating that the all‐solid‐state sodium‐ion battery can efficiently store the electrical energy generated by the TENG. The discharge energy of the all‐solid‐state sodium‐ion battery is determined by integrating the area of the discharge curves (Figure 4e)

(3) $$E_{\mathrm{discharge}}\;=\;\int_{0}^{C}V(C)dC$$

where *C* is the specific discharge capacity of the all‐solid‐state battery and *V* is the voltage.

The energy conversion efficiency (η) is an important parameter for self‐powered systems which can be calculated as the ratio of the discharge energy of the all‐solid‐state sodium‐ion battery to the output energy from the TENG after rectification

(4) $$\eta =E_{\mathrm{discharge}}/E_{\mathrm{output}}\times 100\%$$

When the battery is discharged at a current density of 5 mA g^−1^ and the rotation rate of the TENG is 800 rpm, the energy conversion efficiency reaches 62.3%, indicating that the all‐solid‐state sodium‐ion battery is an effective device to store the pulsed output energy of TENGs. As the TENGs can harvest various kinds of mechanical energy, thus there is great potential for integrating TENGs with the all‐solid‐state sodium‐ion batteries to provide more stable power output sustainably.

All‐solid‐state sodium‐ion batteries with the hexagonal P2 structure Na_0.67_Ni_0.23_Mg_0.1_Mn_0.67_O_2_ as the cathode, metallic sodium as the anode, and SPE film as the electrolyte have been demonstrated. The solid‐state sodium‐ion batteries were charged by galvanostatic mode and by pulse mode with the TENGs, respectively. The all‐solid‐state sodium‐ion battery displays excellent cyclic performance up to 1000 cycles with capacity retention of about 85% even at a high current density of 48 mA g^−1^. When charged by the TENG, the energy conversion efficiency of 62.3% is achieved. The integration of TENGs with the safe and durable all‐solid‐state sodium‐ion batteries is potential for providing more stable power output sustainably.

## Experimental Section

*Synthesis of Materials*: The P2‐type Na_0.67_Ni_0.23_Mg_0.1_Mn_0.67_O_2_ was synthesized by a sol–gel method.^24^ The PFSA‐Na polymer electrolyte with 0.91 mmol g^−1^ was prepared by a solution‐casting method according to previous reports.^25^ Then the PFSA‐Na membrane was immersed in 60 mL EC‐PC (*v*:*v* = 1:1) nonaqueous mixed solvents in the presence of 20 g activated 4 Å molecular sieves in a sealed container for 48 h at room temperature.

*Characterization of Membrane*: Tensile strength was conducted by using a universal mechanical tester (Instron4465, Instron Corp., USA) at room temperature. For each sample, six dumbbell shape specimens of dimension of 75 × 4 × 1 mm^3^ were tested at 100 mm min^−1^.

Impedance spectra were measured with an Electrochemical Workstation (Autolab PGSTAT302, Netherlands) over the frequency range from 0.1 to 10^6^ Hz with amplitude of 10 mV in the temperature range from 25 to 70 °C. The sodium transference number of the polymer electrolyte was evaluated using the Bruce–Vincern–Evans method in a symmetrical sodium coin cell (type 2032), applying a 5 mV DC signal and the current flowing through the cells and the impedance values before and after the polarization was measured.

*Electrode Fabrication and Electrochemical Measurements*: The symmetric Na|polymer electrolyte|Na cell was assembled with a coin‐type cell (R2032) in an argon‐filled glovebox. The cell was galvanostatic charged and discharged in a battery test system (LAND CT2001A) at a current density of 0.2 mA cm^−2^.

The electrochemical properties of the all‐solid‐state sodium batteries were tested with a standard coin cell (CR2032) in an argon‐filled glove box as reported in the literature.^24^ The sodium foil was used as the anode in the cells and the PFSA‐Na membrane swollen with EC‐PC mixed solvents were used as electrolytes and separators. The cathode electrode slurry was mixed by 85% active materials, 10% acetylene black, and 5% polyvinylidene fluoride (PVDF).The slurry was coated on an Al foil and dried in vacuum for 12 h at 120 °C. The cells were galvanostatic charged and discharged in a battery test system (LAND CT2001A).The electrometer (Keithley 6514) in series and electrometer (Keithley 6517) in parallel were used to test the instantaneous voltage and current of cells during the charging process on TENGs.

## Conflict of Interest

The authors declare no conflict of interest.
